# Electrical Phenotyping of Aged Human Mesenchymal Stem Cells Using Dielectrophoresis

**DOI:** 10.3390/mi16040435

**Published:** 2025-04-03

**Authors:** Lexi L. C. Simpkins, Tunglin Tsai, Emmanuel Egun, Tayloria N. G. Adams

**Affiliations:** 1 Department of Chemical and Biomolecular Engineering, University of California Irvine, Irvine, CA 92697, USA; crowelll@uci.edu (L.L.C.S.); tunglit@uci.edu (T.T.); eegun@uci.edu (E.E.); 2 Sue and Bill Gross Stem Cell Research Center, University of California, Irvine, CA 92697, USA; 3 Department of Materials Science Engineering, University of California, Irvine, CA 92697, USA; 4 Department of Biomedical Engineering, University of California, Irvine, CA 92697, USA

**Keywords:** human mesenchymal stem cells (hMSCs), in vitro expansion, cellular aging, membrane capacitance, cytoplasm conductivity

## Abstract

Human mesenchymal stem cells (hMSCs) are widely used in regenerative medicine, but large-scale in vitro expansion alters their function, impacting proliferation and differentiation potential. Currently, a predictive marker to assess these changes is lacking. Here, we used dielectrophoresis (DEP) to characterize the electrical phenotype of hMSCs derived from bone marrow (BM), adipose tissue (AT), and umbilical cord (UC) as they aged in vitro from passage 4 (P4) to passage 9 (P9). The electrical phenotype was defined by the DEP spectra, membrane capacitance, and cytoplasm conductivity. Cell morphology and size, growth characteristics, adipogenic differentiation potential, and osteogenic differentiation potential were assessed alongside label-free biomarker membrane capacitance and cytoplasm conductivity. Differentiation was confirmed by histological staining and RT-qPCR. All hMSCs exhibited typical morphology, though cell size varied, with UC-hMSCs displaying the largest variability across all size metrics. Growth analysis revealed that UC-hMSCs proliferated the fastest. The electrical phenotype varied with cell source and in vitro age, with high passage hMSCs showing noticeable shifts in DEP spectra, membrane capacitance, and cytoplasm conductivity. Correlation analysis revealed that population doubling level (PDL) correlated with membrane capacitance and cytoplasm conductivity, indicating PDL as a more precise marker of in vitro aging than passage number. Additionally, we demonstrate that membrane capacitance correlates with the osteogenic marker COL1A1 and that cytoplasm conductivity correlates with the adipogenic markers ADIPOQ and FABP4, suggesting that DEP-derived electrical properties serve as label-free biomarkers of differentiation potential. While DEP has previously been applied to BM-hMSCs and AT-hMSCs, and more recently to UC-hMSCs, few studies have provided a direct comparison across all three sources or tracked changes across continuous expansion. These findings underscore the utility of DEP as a label-free approach for assessing hMSC aging and function, offering practical applications for optimizing stem cell expansion and stem cell banking in clinical settings.

## 1. Introduction

Human mesenchymal stem cells (hMSCs) have garnered significant attention for their therapeutic potential, as they can repair tissues and reduce inflammation when implanted into damaged or diseased sites [1,2]. HMSCs are multipotent cells capable of differentiating into various tissues, such as adipocytes, osteocytes, and chondrocytes [3]. HMSCs have been used to treat Crohn’s disease [4], graft-versus-host disease [5], and various neurological diseases [6,7]. For therapeutic applications, a large quantity of hMSCs are needed, often necessitating stem cell banking through in vitro expansion of isolated cells [8,9]. Although in vitro expansion increases the number of hMSCs available for therapeutic use, it also accelerates cellular aging, altering their function [7,10]. These functional changes include decreased differentiation potential, decreased proliferation, reduced immunosuppressive capacity, and low migration ability [7].

In addition to functional decline, in vitro expansion also alters the heterogeneity of hMSCs. Since hMSCs consist of diverse subpopulations, prolonged culture can lead to shifts in population dynamics, potentially resulting in the selective loss or dominance of certain subsets [11]. Additionally, variability in donor [12], tissue origin [13], and culture conditions [14] can influence hMSC characteristics. Together, in vitro aging and heterogeneity underscore the need for methods to systematically characterize hMSCs during expansion.

Several biological markers are used to assess hMSCs as they undergo in vitro aging. These include changes in morphology (in vitro aged hMSCs tend to be bigger) [15], proliferation rate, alterations in gene expression profiles, and surface protein expression. Additionally, differentiation potential and the ability to secrete immunomodulatory factors are often used as functional readouts. While these markers provide valuable insights, they typically rely on labor-intensive, endpoint assays, limiting their practicality for real-time assessment. Notably, there is no functional predictive marker to quickly assess hMSC functionality during in vitro aging. Dielectrophoresis (DEP), a label-free electrokinetic technique, offers a rapid and non-invasive alternative for analyzing live cells. Previous studies have demonstrated that DEP can detect unique electrical signatures of hMSCs [16,17] and distinguish between other cell types [18,19], suggesting its potential as a tool for monitoring hMSC aging and function.

DEP utilizes nonuniform electric fields to induce cell movement based on their polarizability [20], enabling the characterization of the cells’ electrical properties. Cells experience a force based on the electric field gradient, causing a difference in force between the cell and suspending medium. The DEP force is governed by the following equation [20],

(1) $$F_{DEP}=2\pi \varepsilon_{0}\varepsilon_{med}R^{3}Re\left[f_{cm}\right]\nabla {E_{rms}}^{2},$$

where $\varepsilon_{0}=8.85\times 10^{-12}$ (F/m) is the vacuum permittivity, $\varepsilon_{med}\;$is the permittivity of the surrounding medium (F/m), R is the cell radius (μm), $Re\left[f_{cm}\right]$ is the real part of the Clausius–Mossotti factor (unitless), and $E_{rms\;}$ is the root mean square of the electric field (V/m). The Clausius–Mossotti factor describes cell movement and is expressed as [20],

(2) $$f_{cm}=\frac{\varepsilon_{cell}^{*}-\varepsilon_{med}^{*}}{\varepsilon_{cell}^{*}+2\;\varepsilon_{med}^{*}},$$

where $\varepsilon_{cell}^{*}$ is the complex permittivity of the cells, and $\varepsilon_{med}^{*}$ is the complex permittivity of the suspending medium. Cells are modeled as single-shell spheres, where $\varepsilon_{cell}^{*}$ is replaced by the effective complex permittivity, $\varepsilon_{cell,\;eff}^{*}$,

(3) $$\varepsilon_{cell,\;eff}^{*}=\varepsilon_{med}^{*}\left[\frac{{\left(\frac{R}{R-d}\right)}^{3}+2(\frac{\varepsilon_{cyto}^{*}-\varepsilon_{mem}^{*}}{\varepsilon_{cyto}^{*}+2\;\varepsilon_{mem}^{*}})}{{\left(\frac{R}{R-d}\right)}^{3}-(\frac{\varepsilon_{cyto}^{*}-\varepsilon_{mem}^{*}}{\varepsilon_{cyto}^{*}+2\;\varepsilon_{mem}^{*}})}\right],$$

where d represents the plasma membrane thickness, $\varepsilon_{cyto}^{*}$ is the complex permittivity of the cell cytoplasm, and $\varepsilon_{mem}^{*}$ is the complex permittivity of the cell membrane. The complex permittivity is expressed as,

(4) $$\varepsilon_{i}^{*}=\varepsilon_{0}\varepsilon_{i}+\frac{\sigma_{i}}{\omega j},$$

where $\varepsilon_{i}$ is the permittivity, and $\sigma_{i}$ is the conductivity of the respective component. The subscript *i* denotes the cell, cytoplasm, membrane, or suspending medium.

Polarized cells have unique behaviors in nonuniform electric fields and, in DEP, will explicitly exhibit either positive DEP, where the cells move towards regions of high electric field strength, or negative DEP, where the cells move away from regions of high electric field strength [21]. At low medium conductivities, within the beta dispersion region, 10^4^–10^6^ Hz, cell polarizability is influenced by the plasma membrane and cytoplasm. At lower frequencies, the plasma membrane acts as an insulator shielding the cell’s interior from the electric field, allowing DEP to probe structural and molecular features of the membrane [22]. At higher frequencies, the electric field penetrates the cell surface, probing the cytoplasmic properties [23]. Thus, DEP measurements can capture changes in the cell membrane and cytoplasm that may reflect functional changes associated with in vitro aging.

Membrane capacitance, $C_{mem}$, is a good label-free functional cell biomarker [16,24] and is a measure of a cell’s ability to store charge [25]; it is defined as:

(5) $$C_{mem}=\sqrt{2}\;\sigma_{med}/2\pi rf_{xo}$$

where $\sigma_{med}$ is the medium conductivity, r is the cell radius, and $f_{xo}$ is the crossover frequency. The crossover frequency is the point at which cells experience no net DEP force, marking a transition from negative to positive DEP (or vice versa). Membrane capacitance is a function of the lower crossover frequency. Cytoplasm conductivity, a promising functional biomarker, refers to a cell’s ability to conduct an electric field and can be estimated by Equation (4). Both of these properties are beneficial in the analysis of stem cells. For instance, membrane capacitance has been used to detect stem cells [26], their fate [27], and heterogeneity [17]. DEP can be used to characterize cells based on their electrical phenotype which encompasses the DEP spectra, membrane capacitance, and cytoplasm conductivity.

In this study, we used DEP to characterize the electrical phenotype of hMSCs from adipose tissue (AT), bone marrow (BM), and umbilical cord (UC) as they aged in vitro, examining correlations between cell source, in vitro expansion, growth characteristics, and differentiation potential. HMSCs were expanded from passage 4 (P4) to passage 9 (P9), and their morphology, size, growth characteristics, and electrical phenotype were tracked. Cells were differentiated into adipocytes and osteoblasts at low and high passages, with differentiation assessed using histological staining and RT-qPCR. Our findings revealed that all hMSCs maintained a typical spindle-like, elongated morphology; however, cell size varied, with UC-hMSCs exhibiting the greatest variability across all size metrics and the fastest proliferation rate. Differences in electrical phenotype became more pronounced with in vitro aging, particularly at higher frequencies, with high passage hMSCs showing noticeable shifts in the DEP spectra, membrane capacitance, and cytoplasm conductivity. Membrane capacitance and cytoplasm conductivity correlated with population doubling level (PDL), underscoring its utility as a more precise marker of in vitro aging than passage number alone. Differentiation potential varied by cell source, with AT-hMSCs exhibiting the highest potential. Across all hMSCs, differentiation declined with in vitro aging. Additionally, the electrical phenotype of hMSCs correlated with the gene expression of differentiated cells. Specifically, membrane capacitance was associated with the osteogenic gene COL1A1, while cytoplasm conductivity was associated with adipogenic genes ADIPOQ and FABP4. Altogether, these findings demonstrate that DEP is a robust technique for characterizing the electrical phenotype of hMSCs from different sources as they undergo in vitro aging, providing membrane capacitance and cytoplasm conductivity as predictive, label-free markers of cell function. This is important for optimizing stem cell expansion and stem cell banking.

## 2. Materials and Methods

### 2.1. Cell Culture

Primary AT-hMSCs and UC-hMSCs (ATCC, Manassas, VA, USA) were cultured in MSC basal media (ATCC, Manassas, VA, USA) supplemented with 2% FBS (ATCC, Manassas, VA, USA), 5 ng/mL FGF-1 (ATCC, Manassas, VA, USA), 5 ng/mL FGF-2, 5 ng/mL EGF (ATCC, Manassas, VA, USA), and 0.1× antibiotic-antimycotic. BM-hMSCs (ATCC, Manassas, VA, USA) were cultured in MSC basal media (ATCC, Manassas, VA, USA) supplemented with 7% FBS (ATCC, Manassas, VA, USA), 15 ng/mL IGF-1 (ATCC, Manassas, VA, USA), 125 pg/mL FGF-2 (ATCC, Manassas, VA, USA), 2.4 mM L-Alanyl-L-Glutamine (ATCC, Manassas, VA, USA), and 0.1× antibiotic-antimycotic (Life Technologies, Carlsbad, CA, USA).

All the hMSCs were cultured in TC-treated T-75 flasks (Thermo Fisher, Waltham, MA, USA) by seeding at 5000 cells/cm^2^ and were passaged at ~80% confluence. During the cell passaging step, the growth medium was aspirated, and the monolayer was briefly rinsed using 1× DPBS (Life Technologies, Carlsbad, CA, USA) before 5 mL of 0.05% trypsin-EDTA (Life Technologies, Carlsbad, CA, USA) was added to dissociate the cells. When most of the cells were dissociated from the growth surface, residual trypsin activity was neutralized with an equal volume of a neutralizing solution consisting of 5% FBS (ATCC, Manassas, VA, USA) in 1X DPBS (Life Technologies, Carlsbad, CA, USA). The dissociated cell suspension was centrifuged at 275× *g* for 5 min and resuspended in the new growth medium, and the number of cells was determined. The process continued to age the cells from P4 to P9.

### 2.2. Cell Morphology, Size, and Growth Analysis

At 80% confluency, adherent hMSCs were imaged at 10X magnification for cell morphology and size analysis. Morphology was assessed by identifying characteristic cell shapes, including elongated and spindle-like forms. Cell size was quantified by measuring Feret’s diameter (i.e., the longest distance across the cell), cell perimeter, and cell spacing. Images of cultured cells were processed in ImageJ (version 1.80), where the scale bar was measured and set for analysis. Measurements were selected in the ‘Analyze’ menu under the ‘Set Measurements’ dialog box, with ‘Feret diameter’ selected for cell length, ‘Perimeter’ for cell perimeter, and ‘Centroid’ for cell spacing. For each parameter, 50 cells were measured across three regions per image, totaling 150 cells per image. One image per cell type was analyzed per experimental run, with three biological repeats, resulting in 450 cells measured per cell type.

To assess hMSC growth characteristics, population doubling (PD), doubling time (DT), and PDL were calculated. During each passage, detached cells were mixed 1:1 with 0.4% Trypan blue (Life Technologies, Carlsbad, CA, USA) and counted on a hemacytometer (Hausser Scientific, Horsham, PA, USA). PD was determined using the following equation, assuming exponential growth,

(6) $$PD=\frac{\ln\left(N_{f}\right)-ln\;(N_{i})}{ln(2)}$$

where *N_f_* is the final cell count (cell count at passaging), and *N_i_* is the initial cell count (cell count at seeding). DT was calculated by dividing PD by the number of days in culture. Finally, PDL was determined by assuming PD=0 at initial passaging and summing all PD values,

(7) $$PDL=\sum_{i=1}^{n}PD\left(Pi\right)$$

where *P_i_* is the passage number.

### 2.3. DEP Characterization

For DEP analysis, the hMSCs were dissociated and resuspended in a low conductivity DEP buffer consisting of 8.5% (*w*/*v*) sucrose and 0.3% (*w*/*v*) D-glucose with conductivity adjusted to 100 μS/cm using RPMI-1640. In brief, the hMSCs’ growth media was aspirated, and the cells were rinsed once with 1× DPBS. Then, 5 mL of 0.05% trypsin-EDTA was added to the T-75 flask and incubated at 37 °C until the cells detached, followed by neutralization with trypsin neutralization buffer. The cell suspension was centrifuged at 275× *g* for 5 min, and the pellet was resuspended in the DEP buffer for counting. The cells were washed in the DEP buffer three times to remove residual ions and debris. After the final wash, the cells were resuspended at a final cell concentration of 1 × 10^6^ cells/mL in the DEP buffer, as determined by counting with a hemacytometer. Images of suspended cells in the hemacytometer were taken using a 20× objective to assess cell size for DEP data analysis.

DEP characterization was performed using the 3DEP analyzer (LabTech, Heathfield, UK) to establish the electrical phenotype of hMSCs. Prior to each experiment, the 3DEP chip was primed with ethanol, followed by four washes with Milli-Q water and four washes with DEP buffer. After chip preparation, 80 µL of the cell suspension was loaded into the chip and covered with an 18 × 18 mm coverslip. Cells were characterized over a frequency range of 2 kHz to 20 MHz using 20 log-linear frequency steps. At least 9 technical replicates were collected per sample to generate the DEP spectra.

Membrane capacitance, cytoplasm conductivity, membrane permittivity, and cytoplasm permittivity of the hMSCs were determined using the core–shell spherical DEP polarization model (Equations (1)–(5)) with iterative nonlinear fitting, as described in our previous publication [17]. The model was fitted to all technical replicates within an independent experiment (n), resulting in a fit to at least 180 data points. The MATLAB R2023b code is publicly available on GitHub (https://github.com/adamstn2/Core-shell-spherical-DEP-polarization-model.git).

### 2.4. In Vitro hMSC Differentiation and Histology

For adipogenic and osteogenic differentiation, the hMSCs were seeded in their respective growth media at 10,000 cells/cm^2^ in TC-treated 12-well plates coated with 200 µg/mL of porcine skin gelatin (Sigma Aldrich, St. Louis, MO, USA). After two days, adipogenic differentiation was initiated by replacing the growth media with differentiation media consisting of 500 µM isobutyl-methylxanthine, 50 µM indomethacin, 100 nM dexamethasone (all from MP Biomedicals, Irvine, CA, USA), and 0.1× Anti-Anti (Life Technologies, Carlsbad, CA, USA) in DMEM-HG (Life Technologies, Carlsbad, CA, USA). For osteogenic differentiation, the media consisted of 50 μM ascorbic acid 2-phosphate (FUJIFILM Wako, Richmond, VA, USA), 10 mM β-glycerophosphate (Alfa Aesar, Haverhill, MA, USA), 100 nM dexamethasone (MP Biomedicals, Irvine, CA, USA), 10% (*v*/*v*) FBS-HI, and 0.1× Anti-Anti (Life Technologies, Carlsbad, CA, USA) in MEM Alpha (Life Technologies, Carlsbad, CA, USA). The differentiation media were replaced every four days until day 21.

After 21 days of differentiation, the media were aspirated, and the cells were fixed with 4% paraformaldehyde for 10 min. To visualize fatty acid deposits formed during adipogenesis, the cells were stained with Oil Red O (ORO). A 0.5% ORO solution was mixed 3:2 with deionized water and allowed to stand for 20 min before sterile filtering. The cells were stained for 10 min and rinsed three times with deionized water to remove residual ORO. To visualize the hydroxyapatite deposits formed during osteogenesis, the fixed cells were stained with 2% Alizarin Red S (ARS) solution for 15 min. To make the ARS staining solution, the ARS was weighed and dissolved in deionized water, and pH adjusted to 4.1–4.3 with 10% ammonium hydroxide. The resulting solution was sterile-filtered and used immediately. After staining, the cells were rinsed three times with deionized water to remove residual ARS.

Following histological staining, the wells were imaged using an inverted microscope (Keyence, Osaka, Japan) at 10× magnification. To ensure comprehensive coverage, 25 images (5-by-5 grid) were captured for each well and stitched together. To quantify ORO and ARS staining, the stitched images were processed with ImageJ. To account for the difference in staining intensity, the RGB color images were converted to 8-bit grayscale, and the staining intensity was quantified by measuring the mean gray value (0–255 scale) using thresholding. Positive staining was determined by calculating the area ratio of positively stained regions over the total image area. A visual workflow of the image analysis process is provided in Supplemental Figure S1.

### 2.5. RT-qPCR

On days 4, 8, 12, and 16, differentiating cell samples were collected and lysed, and total RNA was extracted and purified with the Qiagen RNeasy Mini Plus Kit following the manufacturer’s instructions. Next, cDNA conversion was conducted using the LunaScript RT SuperMix Kit (New England Biolabs, Ipswich, MA, USA) with the appropriate amount of total RNA. Relative gene expression changes were assessed using multiplex qPCR with the Luna Universal Probe qPCR Master Mix (New England Biolabs, Ipswich, MA, USA). ADIPOQ, FABP4, and PPARG were used to quantify adipogenesis, while ALPL, COL1A1, and RUNX2 were used to quantify osteogenesis. All reactions used GAPDH as the internal control, and all probe assays were purchased from Integrated DNA Technologies (Coralville, IA, USA). Each reaction was performed in duplicate wells as technical replicates.

Raw data from Quantstudio 7 (Life Technologies, Carlsbad, CA, USA) were imported into ThermoFisher Cloud (Thermo Fisher Scientific, Waltham, MA, USA) for preliminary quality control and ΔΔCt relative quantification. For differentiation-related genes that were either not expressed or detected at extremely low levels in the control cells, a ΔCt value of 15 was assigned for the generation of heatmaps.

### 2.6. Statistical Analyses

All statistical analyses were performed using GraphPad Prism (version 10.2.0). One-way ANOVA with Tukey’s post hoc test was used for multiple comparisons in cell size measurements and quantification of histological staining. Pearson’s correlation coefficient was used to evaluate relationships between cell growth characteristics (passage number and PDL), properties from the electrical phenotype (membrane capacitance and cytoplasm conductivity), cell size of suspended cells in DEP buffer, and relative gene expression for adipogenesis and osteogenesis. Histological staining quantification analysis was conducted using two-way ANOVA with Tukey’s post hoc test. The number of biological replicates *n* is specified in the figure legends.

## 3. Results

### 3.1. Morphology, Cell Size, and Growth Characteristics of AT-hMSCs, BM-hMSCs, and UC-hMSCs

**Figure 1:**
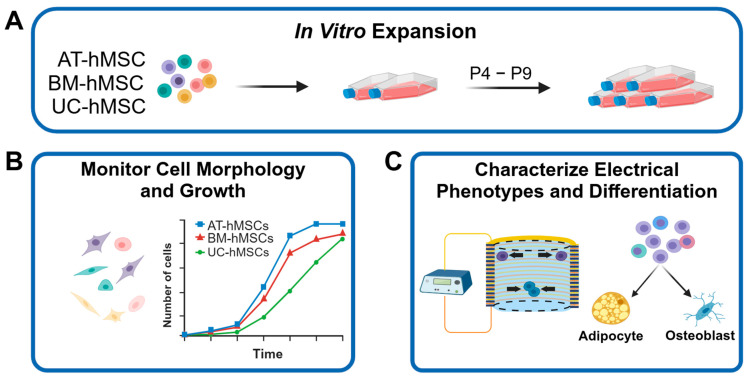
Experimental workflow of hMSC characterization. (**A**) In vitro expansion of AT-hMSCs, BM-hMSCs, and UC-hMSCs from P4 to P9. (**B**) Illustrative representations of cell morphologies and growth. (**C**) Schematic of DEP device used for electrical phenotyping and differentiation of cells into adipocytes and osteoblasts. Figure created in Biorender.com accessed on 1 March 2025.

In this study, we systematically compared three sources of hMSCs, AT-hMSCs, BM-hMSCs, and UC-hMSCs, aged in vitro from P4 to P9, assessing their morphology, cell size, growth characteristics, electrical phenotype, and differentiation potential, as shown in Figure 1. Cell morphology and cell size were examined using phase contrast imaging, growth characteristics were evaluated by tracking cell proliferation over time, and the electrical phenotype was measured using DEP. To assess hMSC function, cells were differentiated into adipocytes and osteoblasts over 21 days.

**Figure 2:**
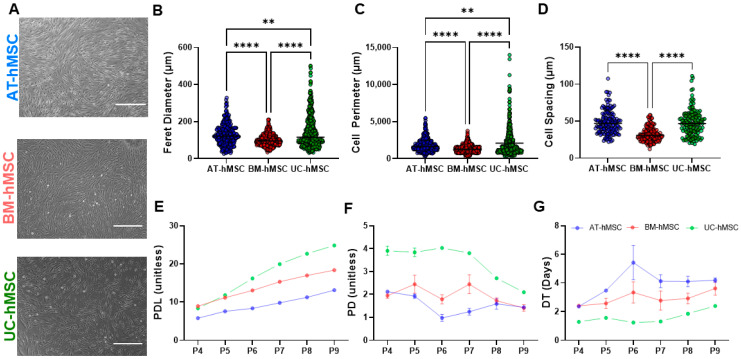
Cell morphology, size, and growth characteristics of AT-hMSCs, BM-hMSCs, and UC-hMSCs. (**A**) Microgram of adherent cells at 10× magnification, scale bar = 400 µm. (**B**) Feret diameter, (**C**) cell perimeter, (**D**) cell spacing, (**E**) PDL, (**F**) PD, and (**G**) DT. The black lines in the dot plots represent the average value. n = 3; ** *p* < 0.01 and **** *p* < 0.0001.

The AT-hMSCs, BM-hMSCs, and UC-hMSCs had fibroblastic morphology (spindle-like, elongated), as shown in Figure 2A. The size of adherent hMSCs was quantified using Feret diameter, cell perimeter, and cell spacing. The AT-hMSCs had the largest Feret diameter compared to the BM-hMSCs (**** < 0.0001) and UC-hMSCs (** < 0.01) (Figure 2B). The UC-hMSCs exhibited a higher standard deviation in the Feret diameter followed by the AT-hMSCs and BM-hMSCs. For cell perimeter, the UC-hMSCs were significantly larger than the AT-hMSCs (** < 0.01) and BM-hMSCs (**** < 0.0001), with the UC-hMSCs also showing the highest standard deviation (Figure 2C). Cell spacing was significantly greater for the UC-hMSCs and AT-hMSCs compared to the BM-hMSCs (**** < 0.0001) (Figure 2D).

With cell culture, differences were observed in the proliferation rates of the hMSCs. To quantitatively assess these differences, we characterized cell growth over in vitro aging by calculating PDL, PD, and DT. The PDL trends in Figure 2E showed that the BM-hMSCs underwent the most population doublings at P4, followed closely by the UC-hMSCs, while the AT-hMSCs experienced the least population doubling. However, the UC-hMSCs had the highest PDL at P5 and remained the highest through P9. In Figure 2F, the UC-hMSCs exhibited the highest PD at each passage, approximately twice that of the AT-hMSCs and BM-hMSCs from P4 to P7. After P7, the PD of the UC-hMSCs declined from 4 PD to ~2 PD. In comparison, the AT-hMSCs and BM-hMSCs exhibited smaller variations in PD from P4 to P9. The DT trends (Figure 2G) increased for all the hMSCs, following the pattern UC-hMSCs < BM-hMSCs < AT-hMSCs.

### 3.2. Electrical Phenotype of AT-hMSCs, BM-hMSCs, and UC-hMSCs

**Figure 3:**
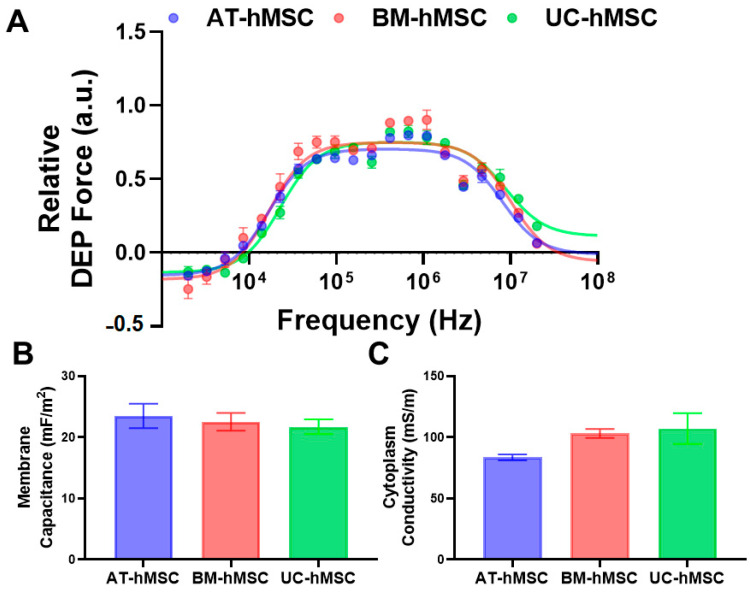
Electrical phenotype of low passage (P4 and P5) AT-hMSCs, BM-hMSCs, and UC-hMSCs. (**A**) DEP spectra fitted with the core–shell spherical polarization model. (**B**) Membrane capacitance and (**C**) cytoplasm conductivity, plotted by cell source. Each dataset represents n = 3.

The growth characteristics may serve as an indicator of differences in the electrical phenotype of hMSCs as well as their differentiation potential. To explore this, we characterized the electrical phenotype of AT-hMSCs, BM-hMSCs, and UC-hMSCs defined by DEP spectra, membrane capacitance, and cytoplasm conductivity. Figure 3A shows the averaged DEP spectra of low passage (P4 and P5) hMSCs. Each curve represents the DEP core–shell spherical polarization model fit to the discrete data points using iterative nonlinear curve fitting, as previously developed in [17]. Descriptively, the DEP spectra showed that the hMSCs experienced negative DEP at frequencies below 10^4^ Hz, with a transition to positive DEP occurring at the crossover frequency around 10^4^ Hz. Following this transition, the DEP spectra reached a maximum between 10^5^ and 10^6^ Hz before gradually decreasing at frequencies above 10^6^ Hz. While the DEP spectra were similar at low frequencies for the AT-hMSCs, BM-hMSCs, and UC-hMSCs, they became discernible at higher frequencies, as emphasized in Supplemental Figure S2. Membrane capacitance and cytoplasm conductivity were extracted from the DEP spectra, as shown in Figure 3B,C, using the modeling approach shown in Figure S3. The membrane capacitance was lowest for the UC-hMSCs and highest for the AT-hMSCs, while the trend for cytoplasm conductivity was the opposite, with the AT-hMSCs having the lowest value and the UC-hMSCs the highest.

**Figure 4:**
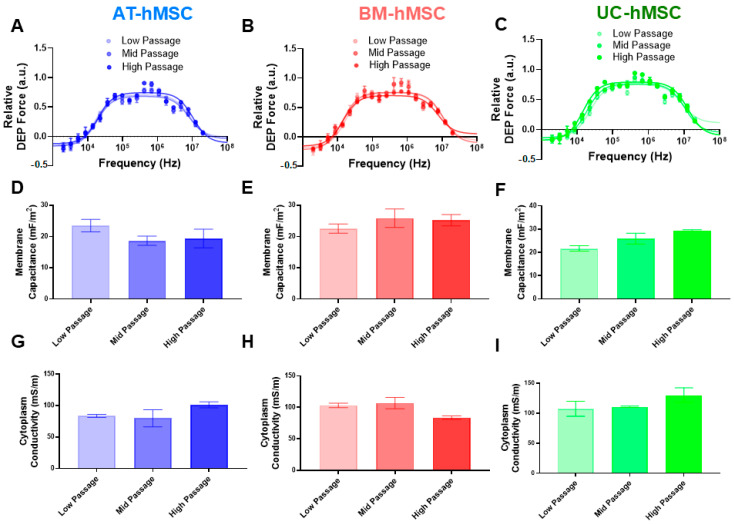
Electrical phenotype of AT-hMSCs, BM-hMSCs, and UC-hMSCs at low (P4 and P5), mid (P6 and P7), and high (P8 and P9) passages. (**A**–**C**) DEP spectra fitted with the core–shell spherical polarization model. (**D**–**F**) Membrane capacitance and (**G**–**I**) cytoplasm conductivity, plotted by cell source. Each dataset represents n = 3.

We expanded the electrical phenotype of the AT-hMSCs, BM-hMSCs, and UC-hMSCs based on in vitro age (Figure 4). The in vitro age was categorized into low (P4 and P5), mid (P6 and P7), and high passage (P8 and P9) groups. The DEP spectra for all the hMSCs retained a consistent shape, exhibiting both negative and positive DEP responses. For the AT-hMSCs, high passage cells were discernible from low and mid passage cells at higher frequencies, with the DEP spectra shifted up (Figure 4A). Correspondingly, membrane capacitance decreased while cytoplasm conductivity increased with in vitro age (Figure 4D,G). For the BM-hMSCs, high passage cells were also discernible at higher frequencies but with the DEP spectra shifted down (Figure 4B). Membrane capacitance increased while cytoplasm conductivity decreased with in vitro age (Figure 4E,H). Lastly, the UC-hMSC high passage cells were discernible across all frequencies, with the DEP spectra shifted up (Figure 4C). In this case, both membrane capacitance and cytoplasm conductivity increased with in vitro age (Figure 4F,I). Supplemental Figure S4 summarizes membrane permittivity and cytoplasm permittivity across all hMSC sources. Membrane permittivity followed a trend similar to membrane capacitance, decreasing with in vitro age for the AT-hMSCs and increasing for the BM-hMSCs and UC-hMSCs. In contrast, cytoplasm permittivity exhibited greater variability across all hMSC sources, with no consistent trend observed. Across all hMSC sources, in vitro aging resulted in observable trends in the DEP spectra, membrane capacitance, and cytoplasm conductivity, but none of these differences reached statistical significance.

**Figure 5:**
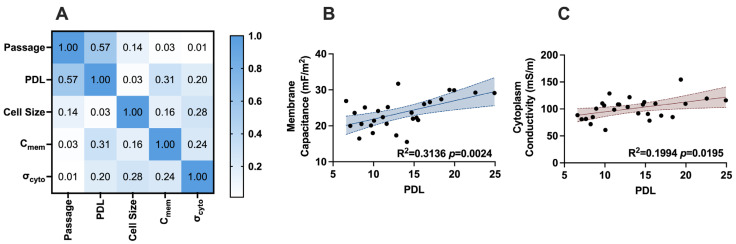
Pearson’s correlation of AT-hMSCs, BM-hMSCs, and UC-hMSCs growth characteristics and electrical properties. (**A**) Heatmap of Pearson’s correlation coefficients (R^2^). *C_mem_* and *σ_cyto_* denote membrane capacitance and cytoplasm conductivity, respectively. (**B**) Linear regression of membrane capacitance and PDL. (**C**) Linear regression cytoplasm conductivity and PDL. Shading in (**B**,**C**) represents 95% confidence interval.

We further explored correlations between in vitro age and the electrical phenotype of AT-hMSCs, BM-hMSCs, and UC-hMSCs. Pearson’s correlation analysis, visualized as a heatmap in Figure 5A, illustrates the relationship between growth characteristics (cell passage number and PDL), cell size (from DEP analysis), and electrical properties (membrane capacitance (*C_mem_*) and cytoplasm conductivity (*σ_cyto_*)). The blue shading in the heatmap represents the coefficient of determination (R^2^), where values along the diagonal are 1, indicating a perfect correlation when a parameter is compared to itself. Among the analyzed parameters, membrane capacitance and cytoplasm conductivity exhibited the strongest correlation with PDL, with R^2^ values of 0.31 and 0.20, respectively. These relationships are further depicted in Figure 5B,C, where linear regression analysis was performed. The correlation between membrane capacitance and PDL was statistically significant (*p* = 0.0024), as was the correlation between cytoplasm conductivity and PDL (*p* = 0.0195).

### 3.3. Differentiation Potential and Gene Expression Analysis of AT-hMSCs, BM-hMSCs, and UC-hMSCs

**Figure 6:**
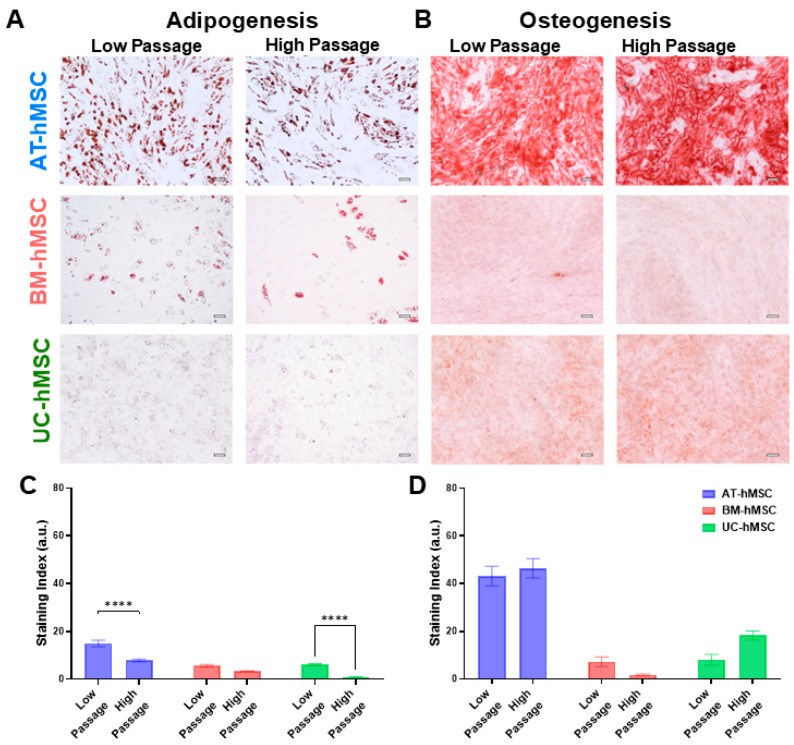
Differentiation of AT-hMSCs, BM-hMSCs, and UC-hMSCs and staining quantification. (**A**) Adipogenesis was assessed with Oil Red O (ORO) at low and high passages. (**B**) Osteogenesis was assessed with Alizarin Red S (ARS) at low and high passages. All cells were visualized with 10× magnification. Scale bar = 100 μm. Quantification of (**C**) ORO stained and (**D**) ARS stained cells at low and high passages. n = 3; **** *p* < 0.0001.

The adipogenesis and osteogenesis potential of the AT-hMSCs, BM-hMSCs, and UC-hMSCs were assessed at low (P4 and P5) and high (P8 and P9) passages to evaluate potential dependencies on in vitro age (Figure 6). Differentiation into adipocytes and osteoblasts was determined using histological stains with ORO and ARS, respectively (Figure 6A,B). Staining intensity was quantified in ImageJ to generate a staining index (Figure 6C,D). Comparing the three sources, the AT-hMSCs exhibited the highest ORO staining at the low and high passages, followed by the BM-hMSCs and UC-hMSCs. Similarly, the AT-hMSCs displayed the highest ARS staining at the low and high passages, followed by the UC-hMSCs and BM-hMSCs. Statistical comparisons between the low and high passages within each hMSC source (e.g., AT-hMSCs low passage vs. AT-hMSCs high passage) are shown with significant differences observed for ORO staining but not for ARS staining. In contrast, comparisons across the hMSC sources (e.g., AT-hMSCs low passage vs. BM-hMSCs low passage) revealed statistically significant differences for ORO and ARS, as detailed in Supplemental Table S1.

**Figure 7:**
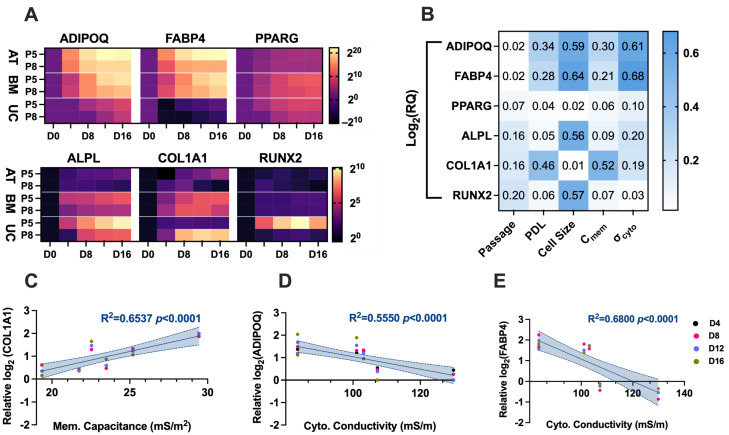
Gene expression and correlation analysis of differentiated AT-hMSCs, BM-hMSCs, and UC-hMSCs. (**A**) Heatmaps of adipogenic and osteogenic expression at low (P5) and high (P8) passages. (**B**) Heatmap of Pearson’s correlation coefficients of (R^2^). *C_mem_* and *σ_cyto_* denote membrane capacitance and cytoplasm conductivity, respectively. (**C**) Linear regression of COL1A1 and membrane capacitance. (**D**) Linear regression of ADIPOQ and cytoplasm conductivity. (**E**) Linear regression of FABP4 and cytoplasm conductivity. Shading in (**C**–**E**) represents a 95% confidence interval.

Gene expression changes during adipogenic and osteogenic differentiation of the AT-hMSCs, BM-hMSCs, and UC-hMSCs were tracked using RT-qPCR. The mean expression levels for the adipogenic and osteogenic markers are presented as heat maps in Figure 7A (top row). During adipogenesis, the AT-hMSCs and BM-hMSCs exhibited an exponential upregulation of ADIPOQ and FABP4 at both low (P5) and high (P8) passages, with expression increasing from Day 4 (D4) to Day 16 (D16), as indicated by the transition from purple to yellow in the heatmap. The UC-hMSCs also showed an upregulation of ADIPOQ at both passages, though to a lesser extent than the AT-hMSCs and BM-hMSCs (purple to magenta shift in the heatmap). Additionally, the UC-hMSCs exhibited low expression of FABP4 at both low and high passages. Interestingly, PPARG was upregulated in the BM-hMSCs and UC-hMSCs, though its increase was less compared to ADIPOQ and FABP4, with expression changes observable from D4 to D16. Overall, the adipogenic gene expression peaked at D16, with the lower passage hMSCs exhibiting a higher degree of upregulation upon differentiation.

For osteogenesis, in Figure 7A bottom row, the overall upregulation of osteogenic genes (ALPL, COL1A1, and RUNX2) was lower than that of adipogenic genes. The BM-hMSCs and UC-hMSCs exhibited exponential upregulation of ALPL and COL1A1 at both low and high passages, with expression increasing from D4 to D16, as indicated by the transition from black to red-pink, magenta, or yellow in the heatmap, except for the low-passage UC-hMSCs. The AT-hMSCs displayed lower upregulation of ALPL and COL1A1 at both passages (black to dark purple shift in the heatmap). Interestingly, COL1A1 expression in the low passage AT-hMSCs and high passage BM-hMSCs peaked between D8 and D12 before slightly decreasing at D16. RUNX2 expression remained low across all hMSC sources, except for the low passage UC-hMSCs, which exhibited an initial upregulation followed by a decline (black to yellow to orange shift in the heatmap). Overall, the lower passage hMSCs showed greater osteogenic gene upregulation upon differentiation.

To investigate potential correlations between differentiation potential, hMSC growth characteristics, and their electrical properties, a Pearson’s correlation analysis was performed (Figure 7B). R^2^ is displayed and visually represented using a blue heatmap. Notably, weak correlations (R^2^ = 0.02 − 0.20) were observed between passage number and adipogenic and osteogenic genes. However, weak (R^2^ = 0.34, 0.28) to moderate (R^2^ = 0.46) correlations were observed between PDL and adipogenic (ADIPOQ and FABP4) and osteogenic (COL1A1) genes, respectively. Moderate correlations (R^2^ = 0.56–0.64) were observed between cell size for DEP analysis and adipogenic (ADIPOQ and FABP4) and osteogenic (ALPL) genes. For electrical properties, membrane capacitance showed weak correlations with adipogenic genes (R^2^ = 0.30, 0.21 for ADIPOQ and FABP4, respectively) but a moderate correlation with the osteogenic gene COL1A1 (R^2^ = 0.52). In contrast, cytoplasm conductivity exhibited moderate correlations with adipogenic genes (R^2^ = 0.61, 0.68 for ADIPOQ and FABP4, respectively) but weak correlations with osteogenic genes (R^2^ = 0.20, 0.19 for ALPL and COL1A1, respectively). Correlations with RUNX2 were displayed but not emphasized due to its low expression levels. The highest correlations involving membrane capacitance and cytoplasm conductivity are further analyzed in Figure 7C–E, where gene expression is normalized to the average expression of all hMSCs analyzed on the same day. Linear regression analysis was performed, with data points color-coded by differentiation day. The best-fit line is shown with a 95% confidence interval shaded. These specific correlations were statistically significant (*p* < 0.0001).

## 4. Discussion

In hMSC transplantation studies and clinical trials, large numbers of cells are required, making stem cell banking an essential component of the process. To meet the demand of 50 million+ cells [28], large-scale in vitro expansion is necessary [29,30]. However, this expansion involves continuous passaging, and it is well documented that cell proliferation and differentiation potential change with passaging due to the dynamic nature of these biological processes [14,31,32]. These changes contribute to inconsistent clinical outcomes [33]. Additionally, the inherent heterogeneity of hMSC populations, which evolves with in vitro aging, further exacerbates variability in clinical trials [32].

Given these challenges, characterizing the electrical phenotype of hMSCs, specifically their DEP spectra, membrane capacitance, cytoplasm conductivity, as they age in vitro may provide a valuable complementary metric of cell function for stem cell banking. Electrical phenotyping with DEP offers a label-free approach to monitor the dynamic changes in cell populations, potentially enhancing quality control and consistency in clinical trials. In this study, we systematically aged AT-hMSCs, BM-hMSCs, and UC-hMSCs, common sources of hMSCs used in clinical applications, from P4 to P9 and examined their morphology, growth characteristics, electrical phenotype, and differentiation potential, as outlined in our workflow (Figure 1).

Microscopic images showed that all sources of hMSCs maintained their spindle-like, elongated shape; however, variability was observed in the Feret diameter, cell perimeter, and cell spacing (Figure 2). While our assessments were completed on low passage hMSCs, Jeske et al. [31] interestingly reported that AT-hMSCs become more elongated and lose their spindle-like shape as passage number increases. Our growth characteristics analysis revealed that UC-hMSCs exhibited the most variability in cell size and the most robust proliferation rates (Figure 2), which is consistent with previous studies in the literature [33,34]. AT-hMSCs and BM-hMSCs displayed relatively similar PD, which gradually declined with increasing in vitro age. Although passage number is often used to define cell age, it provides limited information about cell growth, offering only a measure of how long cells have been in culture. In the context of stem cell banking, monitoring cell age through PDL, PD, and DT is important, as these metrics offer a more nuanced view of cell growth. For example, DT reflects how many cells are produced daily, PD indicates how many cells are generated with each passage, and PDL tracks the total number of cell doublings across passages. Previous studies have shown that passage number (which is implicitly related to DT, PD, and PDL) is inversely correlated with MSC potency in graft versus host disease patients [5]. This decline in potency may be associated with senescence, a process that hMSCs experience as they age [9,35,36]. These differences in in vitro growth point to the inherent heterogeneity among hMSCs derived from different tissue sources and underscore the need for further characterization.

Our electrical phenotyping of hMSCs reveals that UC-hMSCs are more different than AT-hMSCs and BM-hMSCs (Figure 3). Expanded electrical phenotyping incorporating in vitro age shows that hMSCs exhibit greater differences at higher passages, with these differences being more pronounced in cytoplasm conductivity than in membrane capacitance (Figure 4). While these results did not reveal statistically significant differences, the observed patterns suggest subtle changes that may hold biological relevance. Correlations made of data extracted from Figure 2, Figure 3 and Figure 4 show that there is a moderate and statistically significant correlation between PDL and membrane capacitance (R^2^ = 0.3136, *p* = 0.0024), indicating that 31.36% of the variation in membrane capacitance is explained by PDL (Figure 5). Additionally, there is a weak but statistically significant correlation observed between PDL and cytoplasm conductivity (R^2^ = 0.1994, *p* = 0.0195), indicating that approximately 19.94% of the variation in cytoplasm conductivity is explained by PDL (Figure 5). These correlations suggest that PDL is an important factor to consider in the in vitro aging process.

With in vitro aging, the cell membrane becomes more rigid and ordered, and the composition changes [37,38]. Alterations in membrane composition, such as increased cholesterol content or lipid saturation, could influence the activity of ion channels and transporters, thereby affecting ion flux and cytoplasmic ion concentrations [38]. Changes in ion transport with aging may also lead to shifts in cytoplasmic ion concentration, which could contribute to the observed differences in cytoplasm conductivity. These changes align with the membrane hypothesis, which posits that structural and compositional changes in the cell membrane during aging disrupt ion transport and the electrical properties of cells [37]. Our findings highlight the complexity of the electrical phenotype of cells, where variations in the DEP spectra, membrane capacitance, and cytoplasm conductivity may not always result in statistically significant differences when assessed by passage number but can still provide valuable insights when alternative metrics such as PDL are considered.

The differentiation potential of AT-hMSCs, BM-hMSCs, and UC-hMSCs reveals that there are greater differences in the adipogenic and osteogenic potential across the cell source rather than in vitro age (Figure 6 and Figure 7A). However, lower passage hMSCs exhibited a higher degree of adipogenic and osteogenic gene upregulation upon differentiation, suggesting a decline in adipogenic and osteogenic potential with in vitro aging. Compiling data extracted from the electrical phenotype (membrane capacitance and cytoplasm conductivity) and differentiation (gene expression) revealed statistically significant correlations. Specifically, a strong correlation was observed between membrane capacitance and COL1A1 (R^2^ = 0.6537, *p* < 0.0001), indicating that 65.37% of the variation in COL1A1 gene expression is explained by membrane capacitance (Figure 7C). Since membrane capacitance is estimated from the DEP spectra at lower frequencies, where cell polarization is primarily influenced by the cell membrane, it is possible that changes in membrane composition, including glycosylation patterns, contribute to this relationship. Interestingly, the relative abundance of oligomannose and complex N-glycans changes as MSCs undergo osteogenesis [39], which may help explain the observed correlation between membrane capacitance and COL1A1 gene expression. Furthermore, the literature findings implicate glycosylation in the regulation of cell differentiation for neural stem and progenitor cells [40]. There is a moderate correlation between cytoplasm conductivity and ADIPOQ (R^2^ = 0.5550, *p* < 0.0001), indicating that approximately 55.5% of the variation in ADIPOQ gene expression is explained by cytoplasm conductivity (Figure 7D), and a strong correlation between cytoplasm conductivity and FABP4 (R^2^ = 0.6800, *p* < 0.0001), indicating that approximately 68.0% of the variation in FABP4 gene expression is explained by cytoplasm conductivity (Figure 7E). The cytoplasm is a crowded space containing many organelles, with mitochondria being particularly abundant, as evidenced by ultrastructural cell imaging [41,42,43]. The structure of mitochondria is linked to hMSC differentiation potential [44], which may help explain the observed correlation between cytoplasm conductivity and ADIPOQ and FABP4 gene expression. Additionally, studies have shown that DEP can detect intracellular and isolated mitochondria [45,46], further supporting the potential link between cytoplasm conductivity and mitochondrial involvement in hMSC differentiation.

Altogether, our findings indicate that DEP is a robust label-free technique for characterizing the electrical phenotype of hMSCs from different sources and across in vitro aging. By reducing the need for flow cytometry or other label-based bioanalytical techniques, DEP provides a streamlined and non-invasive approach to monitor cell populations. This study suggests that the electrical phenotype of hMSCs, as measured by DEP, is a valuable indicator for determining optimal passage number and cell source for in vitro expansion in clinical applications. Specifically, our results demonstrate that membrane capacitance and cytoplasm conductivity have the potential to reflect hMSCs’ in vitro age and differentiation potential. This is particularly important because methods to assess how in vitro aging impacts cell functionality, such as RNAseq and differentiation assays, are time-consuming, costly, and not practical for routine use in stem cell banking or clinical applications. Advancing more practical and efficient methods would not only enhance quality control but also yield critical insights to improve clinical outcomes. These findings also lay the groundwork foundational DEP-based hMSC sorting strategies to target specific subpopulations (e.g., adipocytes, osteoblasts, and chondrocytes). Given the inherent heterogeneity of hMSCs [17], technologies enabling the efficient label-free sorting would provide significant value to the field of stem cell research.

Previously, Labeed et al. utilized DEP to investigate membrane capacitance as an indicator of neuronal cell fate with the increasing in vitro age of human neural stem and progenitor cells [24]. They found that neuronal cell fate decreased and membrane capacitance increased with in vitro age, providing evidence that the electrical phenotype of stem cells can serve as a predictive marker of cell function. However, their study did not assess cytoplasm conductivity. Many DEP studies focus primarily on characterizing cellular phenotypes but do not specifically consider the impact of in vitro aging on cell function.

Building on this, Yang et al. [14] demonstrated that the phenotype of BM-hMSCs is influenced by in vitro aging and culture conditions. Consistent with our findings, they observed that, regardless of media conditions, PD decreased and DT increased with in vitro age. Additionally, early and late passage MSCs retained their ability to differentiate into adipocytes and osteoblasts. When comparing osteogenic and adipogenic gene expression between early and late passage MSCs, osteogenic gene expression remained similar, whereas adipogenic gene expression decreased by an order of magnitude. These outcomes were consistent. Histological assessments of MSC differentiation further revealed that adipogenic potential was better retained with increasing in vitro age.

The necessary process of in vitro aging for stem cell banking is complex; as such, it is valuable to monitor with multiple predictive indicators of cell function. In the context of previous studies, our research reinforces the utility of DEP in characterizing in vitro aged cells, providing potential predictive markers in the electrical phenotype of cells for clinical applications in stem cell therapy. Our findings emphasize the need for continued exploration of electrical phenotyping as a complementary tool for tracking functional changes in hMSCs over time, particularly in relation to in vitro aging.

Future studies will focus on expanding our sample size by evaluating multiple donors and incorporating clinical samples. Additionally, we plan to increase the number of genes analyzed for adipogenesis and osteogenesis and integrate flow cytometry to assess cell surface protein expression. While this study used passage number to describe in vitro age, future analyses will include PDL as a complementary metric since it provides a more quantitative measurement of cell growth characteristics. DEP-based characterizations reveal how hMSCs change over time and offer a practical approach to optimize expansion conditions, address cell variability, and improve the consistency of hMSC-based therapies. Additionally, DEP could be used to purify cell populations at an optimal in vitro age, further enhancing their clinical potential.

## 5. Conclusions

This study provides a comprehensive evaluation of the electrical phenotype and differentiation potential of in vitro aged AT-hMSCs, BM-hMSCs, and UC-hMSCs, with implications for stem cell banking. By systematically tracking DEP-derived electrical properties across six consecutive passages, we demonstrate that membrane capacitance and cytoplasm conductivity evolve with in vitro aging and correlate with functional metrics, such as differentiation potential and proliferation rates. Notably, our findings suggest that membrane capacitance correlates with osteogenesis (COL1A1 expression), while cytoplasm conductivity correlates with adipogenesis (ADIPOQ and FABP4 expression), positioning these electrical properties as label-free biomarkers of differentiation potential. Additionally, the correlations between membrane capacitance, cytoplasm conductivity, and PDL suggest that PDL is a more precise metric for tracking in vitro aging than passage number alone. These findings reinforce the utility of DEP as a robust, label-free technique for assessing stem cell function, providing a scalable and non-invasive alternative to traditional bioanalytical methods. Given the challenges of assessing in vitro aging, DEP offers a practical solution for routine stem cell monitoring. Developing efficient and scalable methods for tracking hMSC characteristics is essential for improving quality control in stem cell banking and optimizing expansion strategies for clinical applications. DEP-based electrical phenotyping represents a promising tool for addressing these challenges, offering valuable insights into cell function and aging dynamics.

## Data Availability

Data are contained within the article.

## Supplementary Materials

The following supporting information can be downloaded at: https://www.mdpi.com/article/10.3390/mi16040435/s1, Figure S1: Workflow for quantifying histological stains in ImageJ; Figure S2: DEP analysis of higher frequency range of AT-hMSC, BM-hMSC, and UC-hMSC. Black boxes indicate differences in the DEP spectra between cell sources; Figure S3: Representation of the core–shell spherical DEP polarization modeling approach. The DEP spectra are shown as discrete data points for AT-hMSCs, BM-hMSCs, and UC-hMSCs at low passage (P4 and P5) for a single independent experiment (~9 technical replicates); Figure S4: (A) Membrane permittivity and (B) cytoplasm permittivity of AT-hMSCs, BM-hMSCs, and UC-hMSCs at low (P4 and P5), mid (P6 and P7), and high (P8 and P9) passages; Table S1: Statistical significance of different comparisons for histological stains. Each cell source is compared at either low passage or high passage. n = 3; *** *p* < 0.001 and *** *p* < 0.0001.
