# Supplementary material for: Electrical Phenotyping of Aged Human Mesenchymal Stem Cells Using Dielectrophoresis
Source: Micromachines (Basel). 2025 Apr 3;16(4):435. doi: 10.3390/mi16040435 (PMC12029641; doi:10.3390/mi16040435)
Supplement: Supplementary file 1 [file micromachines-16-00435-s001.zip › micromachines-3533618-supplementary.pdf]

Electrical Phenotyping of Aged Human Mesenchymal Stem Cells Using Dielectrophoresis

Lexi L. C. Simpkins [1,2], Tunglin Tsai [1,2], Emmanuel Egun [1,2] and Tayloria N. G. Adams [1,2,3,4,*]

[1] Department of Chemical and Biomolecular Engineering, University of California Irvine, Irvine, CA 92697, USA
[2] Sue and Bill Gross Stem Cell Research Center, University of California, Irvine, CA 92697, USA
[3] Department of Materials Science Engineering, University of California, Irvine, CA 92697, USA
[4] Department of Biomedical Engineering, University of California, Irvine, CA 92697, USA
* Correspondence: tayloria@uci.edu

1. Supplemental Information

The workflow for quantifying histological stains for adipogenesis and osteogenesis differentiation using ImageJ. Images were imported into ImageJ; converted to 8-bit grayscale and the mean gray value was selected in the analyze tab. In the process tab, thresholding was utilized to adjust the highlighting of the sample image. The threshold remained the same per sample and changed when using a different sample condition. The mean gray value was calculated and analyzed.

1. First the image is imported into ImageJ.

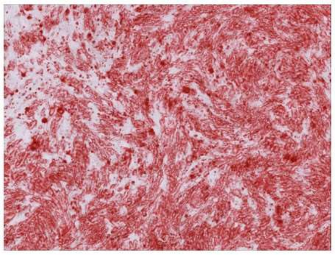

2. The image is converted to 8-bit.

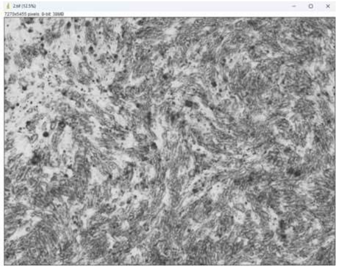

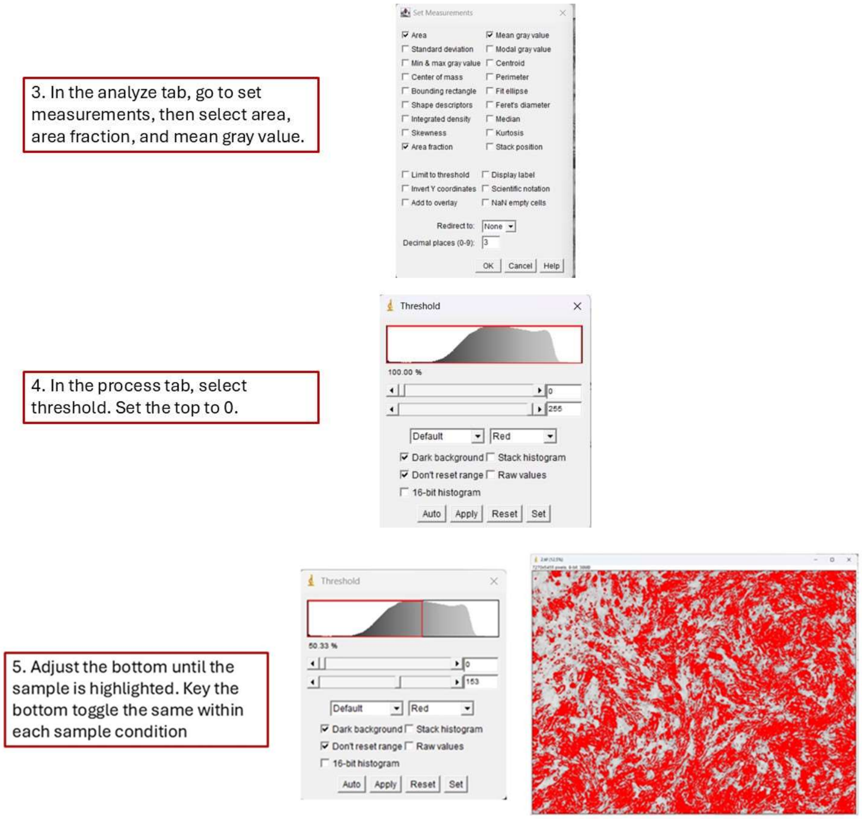

Figure S1. Workflow for quantifying histological stains in ImageJ.

DEP was used to characterize the electrical phenotype of AT-hMSCs, BM-hMSCs, and UC-hMSCs. The spectra data in Figure S2 highlight the frequency range of 10^6 to 10^8 Hz, showing that differences between hMSC sources become more pronounced at higher frequencies. Black boxes emphasize these distinctions.

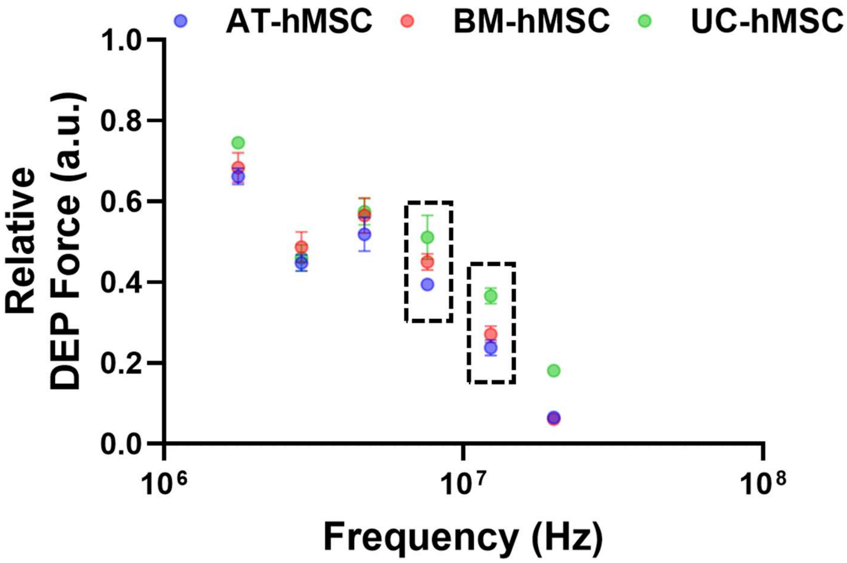

Figure S2. DEP analysis of higher frequency range of AT-hMSC, BM-hMSC, UC-hMSC. Black boxes indicate where there are differences in the DEP spectra between cell sources.

We modeled the electrical properties from the DEP spectra using MATLAB by applying the core-shell spherical DEP polarization model with nonlinear fitting to experimental data. We fit the model to all technical replicates within a single independent experiment. This approach enabled us to determine membrane capacitance, cytoplasm conductivity, membrane permittivity, and cytoplasm permittivity. Figure S3 illustrates our modeling process for a representative dataset of low passage hMSCs, which included ~9 technical replicates per source of hMSCs (n = 1). The red curve in the figure represents the model.

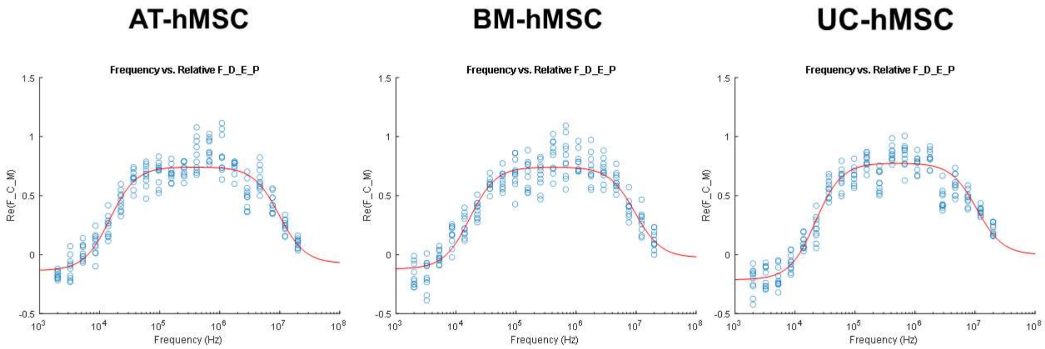

Figure S3. Representation of the core-shell spherical DEP polarization modeling approach. The DEP spectra are shown as discrete data points for AT-hMSCs, BM-hMSCs, and UC-hMSCs at low passage (P4 and P5) for a single independent experiment (~9 technical replicates).

In addition to obtaining the membrane capacitance and cytoplasm conductivity, the membrane permittivity and cytoplasm permittivity were also determined, Figure S4. Membrane permittivity follows a trend similar to membrane capacitance, decreasing with in vitro age for AT-hMSCs and increasing with in vitro age for BM-hMSCs and UC-hMSCs. In contrast, cytoplasm permittivity exhibited greater variability across all hMSCs, with no consistent trend observed.

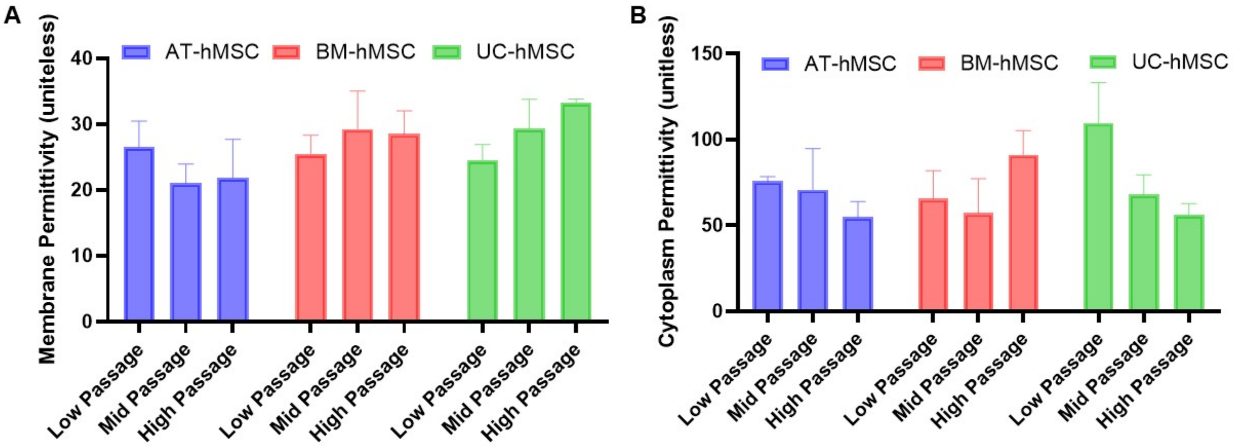

Figure S4. (A) Membrane permittivity and (B) cytoplasm permittivity of AT-hMSCs, BM-hMSCs, and UC-hMSCs at low (P4 and P5), mid (P6 and P7), and high (P8 and P9) passages.

Table S1 shows the statistical significance comparing cell sources to low and high passage after histological staining for adipogenesis and osteogenesis.

Table S1. Statistical significance of different comparisons for histological stains. Each cell source is compared at either low passage or high passage. n = 3; *** $p<0.001$ and **** $p<0.0001$.

Histological Staining	Comparison	Statistical Significance
Adipogenesis	AT-hMSC Low Passage vs. BM-hMSC Low passage	****
	AT-hMSC Low passage vs. UC-hMSC Low passage	****
	AT-hMSC High Passage vs. BM-hMSC High Passage	***
	AT-hMSC High Passage vs. UC-hMSC High Passage	****
Osteogenesis	AT-hMSC Low Passage vs. BM-hMSC Low Passage	****
	AT-hMSC Low Passage vs. UC-hMSC Low Passage	****
	AT-hMSC High Passage vs. BM-hMSC High Passage	****
	AT-hMSC High Passage vs UC-hMSC High Passage	****
